# Single arm, phase two study of low-dose metronomic eribulin in metastatic breast cancer

**DOI:** 10.1007/s10549-021-06175-x

**Published:** 2021-04-02

**Authors:** Pavani Chalasani, Kiah Farr, Vicky Wu, Isaac Jenkins, Alex Liu, Stephanie Parker, Vijayakrishna K. Gadi, Jennifer Specht, Hannah Linden

**Affiliations:** 1 University of Arizona Cancer Center, 3838 N Campbell Ave, Tucson, AZ 85719 USA; 2 College of Medicine, University of Arizona, Tucson, AZ USA; 3 University of Washington, Seattle, WA USA; 4 Fred Hutchinson Cancer Research Center, Seattle, WA USA; 5 Seattle Cancer Care Alliance Evergreen, Kirkland, WA USA; 6 University of Ilinois at Chicago, Chicago, IL USA

## Abstract

**Background:**

Treatment options for metastatic breast cancer (MBC) refractory to anthracyclines and taxanes are limited. In a phase III trial, eribulin demonstrated a significant improvement in overall survival compared to treatment of physician’s choice, but had limited tolerability because of neutropenia and peripheral neuropathy. Based on prior studies of alternative treatment schedules with other therapies, we hypothesized that a low-dose metronomic schedule of eribulin would permit patients to remain on treatment more consistently without treatment delays, resulting in longer time to progression, and improved toxicity profile.

**Methods:**

We conducted a multi-site single arm, phase II trial patients with MBC. All patients were treated with metronomic eribulin (0.9 mg/m^2^ administered intravenously on days 1, 8, and 15 of a 28-day cycle.) Treatment was continued until the patient developed disease progression, unacceptable toxicity, or chose to stop the study. Patients must have had prior taxane exposure. The primary endpoint was progression-free survival. Secondary end points were overall survival, response rate, and clinical benefit rate. Exploratory biomarkers were performed to analyze change in levels of circulating endothelial cells (CECs), circulating endothelial precursors, and carbonic anhydrase IX (CAIX) with response to therapy.

**Findings:**

We consented 86 patients and 59 were evaluable for final analysis. Median age was 59 years; 78% had HER2 negative tumors. The median progression-free survival (PFS) was 3.5 months with overall survival (OS) of 14.3 months. Objective response rate was 15% with clinical benefit rate of 48%. Reported grade 3 neutropenia and peripheral neuropathy were 18% and 5%, respectively. Treatment discontinuation due to toxicity was seen in 3% of patients.

**Interpretation:**

Metronomic weekly low-dose eribulin is an active and tolerable regimen with significantly less myelosuppression, alopecia, and peripheral neuropathy than is seen with the approved dose and schedule, allowing longer duration of use and disease control, with similar outcomes compared to the standard dose regimen.

**Supplementary Information:**

The online version contains supplementary material available at 10.1007/s10549-021-06175-x.

## Introduction

Breast cancer is the most frequently diagnosed cancer in women in the United States and the leading cause of cancer-related deaths in women worldwide [1]. With an increasing armamentarium of therapeutics, patients are living longer with MBC. As the majority of patients with MBC cannot be cured, treatment duration is longer and optimizing treatments to ensure quality of life (QOL) is essential [2]. For hormone receptor positive human epidermal growth factor receptor negative (HR+/HER2−ve) MBC, newer targeted oral treatments are available that have delayed the use of parenteral chemotherapy. However, eventually the majority of these patients will progress and begin chemotherapy. For HER2+ve MBC, sequential single agent chemotherapy in synergy with targeted HER2 therapy is standard treatment. For triple negative MBC, chemotherapy is the mainstay of treatment. NCCN guidelines list over 15 single agent chemotherapy agents and recommend sequential single agent therapy over combination chemotherapy, except in select patients with high tumor burden or rapidly progressing disease and visceral crisis, due to better QOL [3].

In 2010, the Food and Drug Administration approved eribulin mesylate, a non-taxane microtubule dynamics inhibitor, for the treatment of patients with MBC who have previously received at least two chemotherapeutic regimens including an anthracycline and a taxane. The approval came after the pivotal phase III trial (EMBRACE), comparing eribulin administered at 1.4 mg/m^2^ on days 1 and 8 of a 21-day cycle to physician’s choice of salvage therapy [4]. In a major advance in treatment for MBC, overall survival, the primary endpoint, was significantly improved from 9.3 to 13.1 months in patients receiving eribulin. There was a longer but not significant difference in progression-free survival (3.7 vs 2.2 months favoring eribulin treatment arm), and the response rate was higher for eribulin (12.2% vs. 4.7%, *p* = 0.002). However, there were significant adverse effects (AEs) associated with the dose and schedule, including neutropenia (52% all grades and 45% grade 3 or 4) and peripheral neuropathy (35% all grades and 8% grade 3 or 4). Published real-world data and additional clinical trials for eribulin show similar results of AE’s: neutropenia (47–54%), asthenia (15–54%), peripheral neuropathy (24–35%) [5–8]. In addition to supportive care, neutropenia and peripheral neuropathy are generally managed by dose delays or dose reductions, which occur in a significant proportion of patients (30–50%) [4, 9].

Based on the mechanism of action and side effect profile, we hypothesized that the dose and schedule of eribulin could be optimized to reduce AEs. In addition to the direct effects on the tumor cells, lower and more frequent doses of chemotherapy are shown to have an anti-angiogenic effect on endothelial cells in the tumor vasculature [10]. The history of other effective and tolerable agents including paclitaxel, capecitabine, and vinorelbine suggests that a low-dose metronomic schedule of eribulin would be effective. A similar approach was used to optimize metronomic low-dose capecitabine without sacrificing efficacy [11]. Based on available literature, smaller and more frequent doses of eribulin should result in consistent drug delivery, decrease in corresponding side effects, and an increase in tolerability, ultimately resulting in a longer time to progression (TTP) and higher QOL during therapy. Based on the phase I studies of eribulin, which showed efficacy of 0.9 mg/m^2^, we proposed a dose of 0.9 mg/m^2^ on days 1, 8, and 15 of a 28-day cycle as an alternative metronomic schedule in our phase II trial. This dosing schedule was chosen with hopes to avert neutropenia, neuropathy, and alopecia in these patients without compromising efficacy.

## Methods

### Study design and patients

We conducted an open-label, multi-center, single arm phase II study of metronomic eribulin in patients with MBC whose disease has progressed following at least one prior regimen of chemotherapy in the metastatic setting. Main inclusion criteria were prior taxane exposure (in adjuvant, neoadjuvant or metastatic setting), Eastern Cooperative Oncology Group (ECOG) performance status of 0–2; evaluable disease per Response Evaluation Criteria in Solid Tumors (RECIST) 1.1 criteria; adequate bone marrow, renal, and liver function; and a life expectancy of 12 weeks or more. Key exclusion criteria were: prior treatment with eribulin, more than 6 chemotherapy regimens in the metastatic setting, active central nervous system metastases (CNS) (patients with stable CNS metastases were allowed), active secondary primary malignancy, pre-existing baseline peripheral neuropathy of grade 2 or higher. Other anti-tumor systemic therapy was not allowed other than concurrent trastuzumab for HER2+ve cancers and denosumab or bisphosphonates (for metastatic bone disease). Growth factor support and transfusions were permitted as clinically indicated.

All patients provided written informed consent and the study was approved by the ethics committee at University of Arizona and University of Washington. The trial was registered on clinicaltrials.gov (NCT01908101). Data safety and monitoring committee at the Fred Hutchinson/University of Washington Cancer Consortium/ Seattle Cancer Care Alliance Network office oversaw the data and safety monitoring for this study.

### Intervention

Patients received eribulin mesylate administered intravenously at 0.9 mg/m^2^ on days 1, 8, and 15 of a 28-day cycle. Treatment continued until the patient developed disease progression, unacceptable toxicity, or patient requested to stop the study treatment. Grade 3 or 4 toxicities were managed with dose reduction and patients requiring a delay in study treatment of greater than 3 weeks or greater than 3 dose reductions were discontinued from study treatment. Upto three dose reductions for grade 3 or higher toxicities were allowed on the protocol: 0.7 mg/m^2^, 0.6 mg/m^2^, 0.5 mg/m^2^. Patients who continued to have occurrence of grade 3 or higher toxicity at the lowest dose level of 0.5 mg/m^2^ were taken off study.

Our hypothesis was that lower metronomic dose of eribulin will be well tolerated with similar efficacy and less toxicity than standard dose eribulin. The primary objective of this phase II trial was to assess PFS. Secondary objectives included assessment of OS, response rate, clinical benefit rate and the incidence of grade 3 or 4 neutropenia, peripheral neuropathy, and alopecia. Exploratory objective included assessing change in levels of circulating endothelial cells precursors (CEPs), CECs, apoptotic CECs and CAIX levels. Prior reports have shown these levels increase with response to antiangiogenic or metronomic therapies [12–15]. We hypothesized that our proposed metronomic dose of eribulin will have antiangiogenic effects and sought to assess it by measuring changes in these biomarker levels with treatment.

### Statistical analysis

Progression-free survival was defined as the time from study enrollment until earliest date of disease progression or death. PFS was censored at time of last radiographic assessment for patients who discontinued the study for reasons other than disease progression. A sample size of 60 evaluable patients was estimated to provide 99% power for the lower bound of the confidence interval to be greater than 2.2 months (the median PFS in the treatment by physician choice arm of the EMBRACE trial), assuming that the true median PFS for the eribulin arm of the EMBRACE trial was 3.7 months. All patients underwent tumor assessment by computed tomography (CT) every 12 weeks. Tumor response assessment was per RECIST 1.1. Clinical benefit rate was measured as the duration of complete or partial response (CR or PR) or stable disease over 6 months. Safety was assessed according to National Cancer Institute Common Terminology Criteria for Adverse Events (CTCAE) version 4.0. Primary analysis of PFS was performed on per protocol population (defined as patients who completed at least 1 cycle of treatment).

### Correlative studies

#### Sample collection

Blood was collected from patients at baseline and prior to cycles 2, 4, and 6 of chemotherapy using acid citrate dextrose (ACD) as an anticoagulant.

For flow cytometry, blood was processed within 24 h of collection. Four-color flow cytometry was used to quantify CECs, circulating endothelial progenitor cells (CEPs), and apoptotic CECs from breast cancer patients treated with eribulin. We have previously published this method to analyze CECs, CEPs, and apoptotic CECs [15]. Blood cells were stained with monoclonal antibodies, lysed, then analyzed on the BD Accuri C6 Plus flow cytometer (BD Biosciences, San Jose, CA). The monoclonal antibodies used to enumerate CECs and CEPs were anti-CD31 (an endothelial cell marker), CD45 (used to exclude haematopoietic cells), CD133 (an endothelial precursor marker), and LDS751 (a nucleic acid marker used to exclude aggregated platelets). Apoptotic CECs were processed similarly to the above with different markers; in addition to CD31 and CD45, CD41 was used to exclude platelets and 7-aminoactinomycin D was used as an apoptotic cell marker. CECs were defined as LDS751^+^CD31^+^CD45^−^CD133^−^ or 7-AAD^−^CD31^+^CD45^−^CD41^−^, CEPs were defined as LDS751^+^CD31^+^CD45^−^CD133^+^, and apoptotic CECs were defined as 7-AAD^+^CD31^+^CD45^−^CD41^−^. The BD Accuri C6 plus software was used for analysis, and analysis gates excluded platelets, debris, and haematopoietic cells. BD Trucount Tubes (BD Biosciences, San Jose, CA) were used to determine absolute CEC and CEP counts. The number of CECs or CEPs per microliter was calculated as follows: (CEC or CEP count/detected bead count) × (total bead count/100). Percent apoptotic CECs were calculated by dividing the apoptotic CEC count by the CEC count.

For CAIX levels, plasma was collected from the ACD tubes and stored at −80 °C for batch analysis. Quantification was performed using an enzyme-linked immunosorbent assay (ELISA) kit (Quantikine Human CAIX/CA9 Immunoassay, R&D Systems) as we published previously [14]. Plasma CAIX mean, median, and range were determined at each time point.

For exploratory biomarker analysis, patients were divided into two groups based on their baseline values. Assigning a median level cutpoint provided the optimum power (assuming a continuous effect across the spectrum of values), and would be easier to interpret and avoid testing with multiple cutpoints/categories. The differences between the groups were compared and statistical significance was determined by paired *t*-test analysis with *p* values ≤ 0.05 considered significant [13].

## Results

**Fig. 1:**
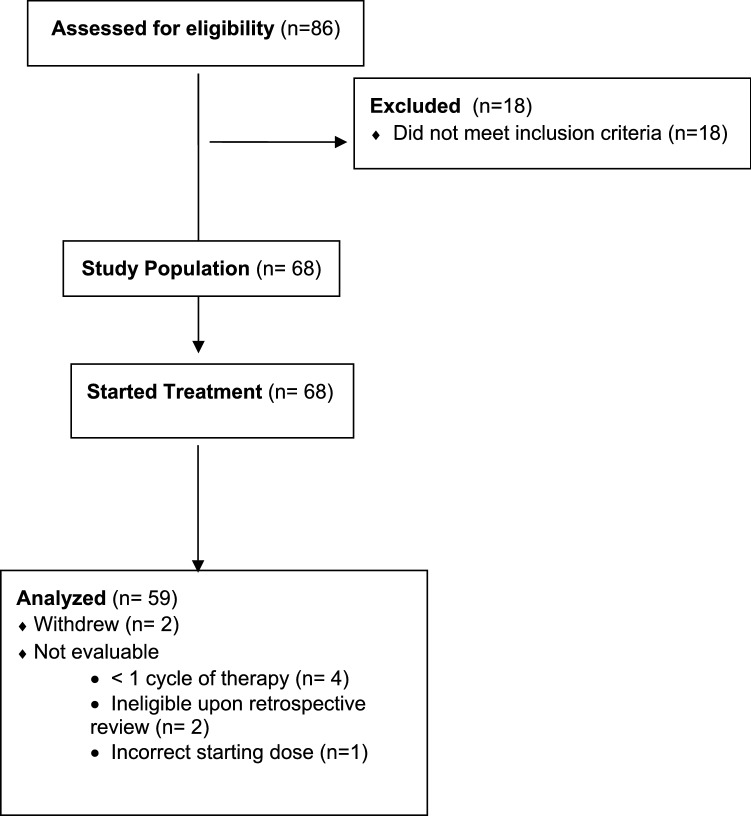
Consort diagram

From 01/2014 to 04/2018, 86 patients were enrolled in this study (Fig. 1). Eighteen patients did not meet eligibility criteria and 68 started on treatment. Two patients withdrew while on study and seven were not evaluable (4 had less than 1 cycle of therapy, 2 were ineligible on retrospective review of their charts as they were determined to have other tumor types and 1 had incorrect starting dose). Baseline demographics of the evaluable population are described in Table 1. The majority of patients were women (99%) and all patients had prior exposure to taxane; the median number of chemotherapy regimens in the metastatic setting was 4 (range 1–6). In addition to taxane, the majority of patients had prior exposure to capecitabine (80%) and anthracycline (66.7%).

**Table 1:** Demographics

Demographics	*n* = 59
Median age (range)	59 (34–83)
Race	
White	49 (83%)
Black	4 (7%)
Asian/pacific Islander	4(7%)
American Indian/Alaskan native	2 (3%)
Number of prior chemotherapy regimens	
1	1 (2%)
2	10 (17%)
3	15 (25%)
4	17 (29%)
5	10 (17%)
6	6 (10%)
Median (range)	4 (1–7)
Prior chemotherapy	
Taxanes	59 (100%)
Anthracyclines	40 (67%)
Capecitabine	48 (80%)
Number of previous hormonal regimens	
0	16(27%)
1	5(8%)
2	12 (20%)
3	7(12%)
≥ 4	19(32%)
ECOG performance status	
0	25 (42%)
1	25 (42%)
Unknown	9 (15%)
HER2 status	
Positive	13 (22%)
Negative	46 (78%)
ER and PR status	
ER and/or PR positive	44 (75%)
ER and PR negative	15 (25%)

**Fig. 2:**
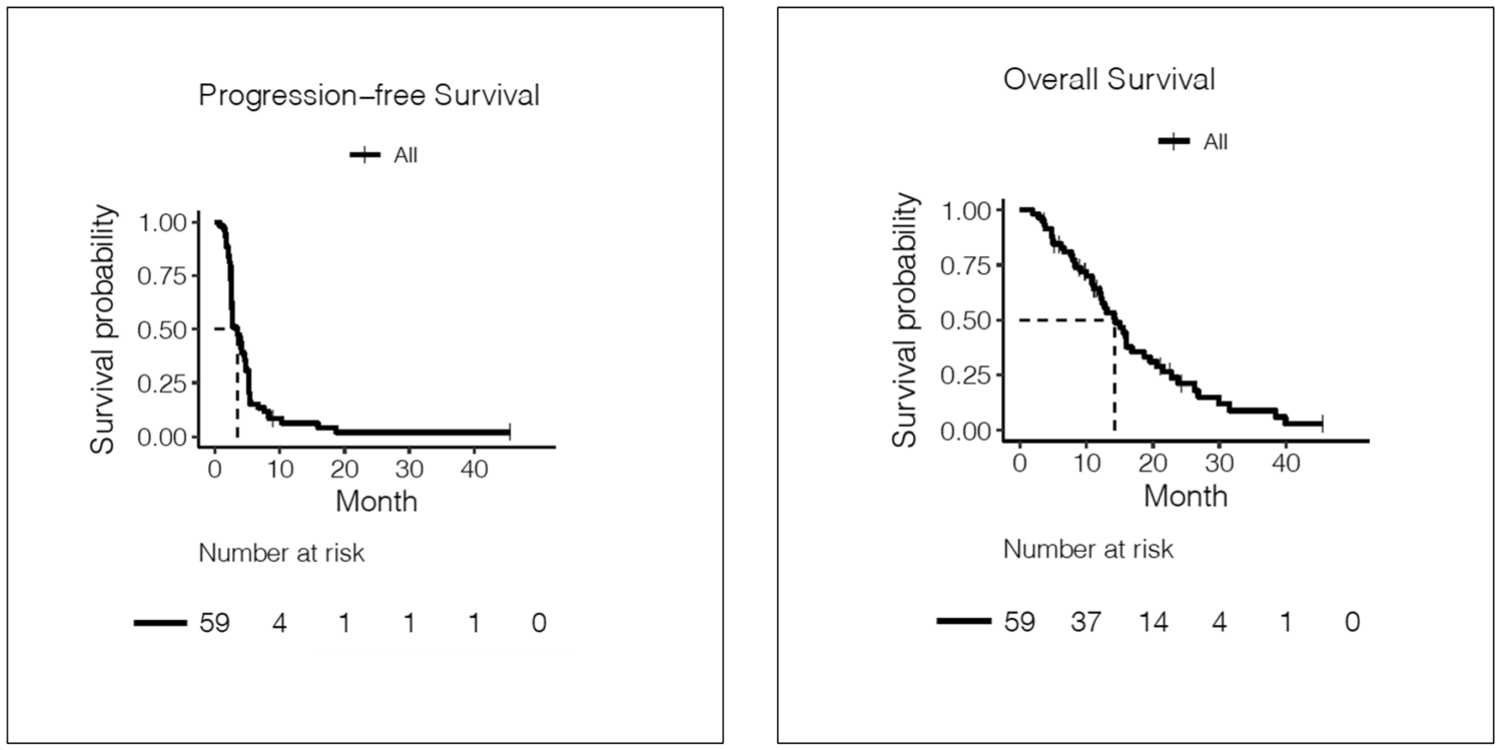
Kaplan–Meier curves for progression-free survival and overall survival

Objective response was seen in 9 patients with 1 patient achieving a CR (Table 2). Objective response in ER+/HER2−ve, triple negative and HER2+ve was 12%, 16.7%, and 23%, respectively. Clinical benefit rate was reported in 49% of patients. The median PFS for metronomic eribulin was 3.5 months (95% CI 2.6–4.8 months) and median OS was 14.3 months (95% CI (12.2–18.7 months) (Fig. 2).

**Table 2:** Efficacy assessments for metronomic eribulin dosing schedule

Efficacy assessment	
Progression-free survival	
Median (months)	3.5
95% Confidence interval	2.6–4.8
Overall survival	
Median (months)	14.3
95% Confidence interval	12.2–18.7

Best overall tumor response	*n*
Complete response	1 (2%)
Partial response	8 (14%)
Stable disease	19 (33%)
Progressive disease	30 (52%)
Unknown	1 (2%)
Objective response rate (*n*/total)^*^	9/59
Clinical benefit rate (*n*/total)^*^	28/59
Clinical benefit rate by tumor subtype (*n*/total)	
HR+ve/HER2−ve	18/34
TNBC	4/12
HER2+ve	6/13

Number of eribulin cycles	*n*
Median	4 (1–15)

*HR* hormone receptor positive, *HER2* human epidermal growth factor receptor 2, *TNBC* triple negative breast cancer

*n = number of patients

Adverse events (AEs) were reported in 95% patients. The most common AE was neutropenia. Grade 3 or 4 neutropenia was reported in 11 patients. Serious adverse events (SAEs) were reported in 2 patients (1 had a myocardial infarction and another had sepsis) and were considered not related to the study drug. Adverse events leading to therapy discontinuation occurred in 2 patients (both due to peripheral neuropathy). Dose delays and reductions were undertaken in 14 and 13 patients, respectively. In patients who did not start with grade 3 or 4 alopecia, there was no worsening of their alopecia grade on our study. Table 3 lists all the grade 3 and 4 study drug-related AEs. (Please refer to supplementary files for all AE data.)

**Table 3:** Grade 3 and 4 adverse events related to study drug

Toxicity	Grade 3		Grade 4	
	*N*	%	*N*	%
Neutropenia	7	12	4	7
Leucopenia	4	7	0	0
Anemia	1	2	0	0
Thrombocytopenia	1	2	0	0
Alopecia	1	2	0	0
Asthenia/fatigue	3	5	0	0
Peripheral neuropathy	3	5	0	0

*N summarizes the number of patients who had adverse events in the study (total evaluable patients = 59)

### Correlative studies

**Fig. 3:**
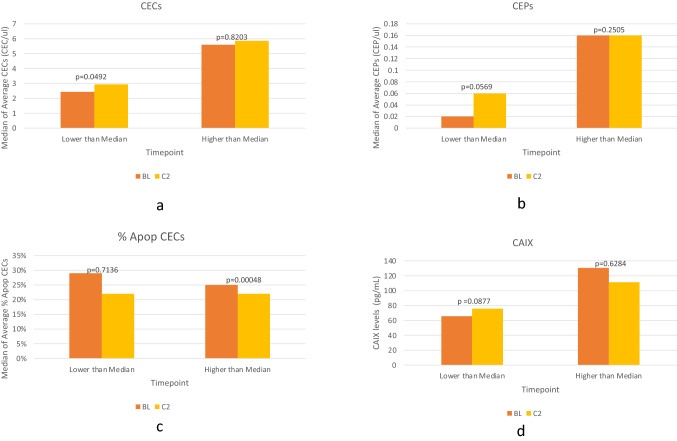
Correlative data showing change in biomarker levels between baseline and cycle 2

We had baseline levels for CECs, CEPs and apoptotic CECs in 59 patients and baseline CAIX levels in 61 patients. We had attrition on the study samples being collected over time. Data on the mean, median, and range for those biomarkers is provided in the supplementary files. Comparison between paired samples was performed between baseline and prior to cycle 2 (Fig. 3). In our patients with lower CEC levels than median at baseline, a significant increase in total CECs was observed in response to treatment, increasing from 2.4 at baseline to 4.3 CEC/μL. For patients with a higher baseline than the median level, there was no significant change (6.4 vs 6.2 CEC/μL). Similar findings were noted with CEPs sub-groups; lower than median group levels increased from 0.02 to 0.09 CEP/μL and higher than median groups increased from 0.21 to 0.36 CEP/μL. Conversely, for apoptotic CECs there was decrease in the levels in both groups with the higher than median group reaching statistical significance. Even though there was no significant difference in change of CAIX levels among both groups, the lower than median sub-group of CAIX levels increased with therapy (65.7–75.8 pg/mL, *p* = 0.08), while it decreased in value in the higher than median group (130.5–111.3 pg/mL) [16–20].

## Discussion

In our single arm, multi-institutional study we demonstrated that a lower dose and alternative schedule (3 weeks on followed by 1 week off) of eribulin is less toxic (with less neutropenia and neuropathy) and has similar efficacy compared to the standard dosing and schedule. Table 4 summarizes the efficacy and toxicity of metronomic dosing compared to those reported in the EMBRACE trial. Our study’s primary end point, PFS, was 3.5 months, which is similar to 3.7 months reported in EMBRACE, and demonstrates efficacy of our low-dose metronomic dosing schedule. Response rate was also demonstrably effective at 15% and 12% for our study and EMBRACE, respectively. Overall survival in EMBRACE was 13.1 months and 15.1 months in our study. These results affirm our original hypothesis that metronomic dosing, as defined by our methods, did not compromise efficacy of treatment of MBC.

**Table 4:** Comparison of efficacy and AEs between low-dose metronomic eribulin and EMBRACE

Efficacy parameter	Metronomic	EMBRACE
PFS (months)	3.5	3.7
OS (months)	14.3	13.1
Response rate	16%	12%
Clinical benefit rate	48%	23%
AEs		
Neutropenia^a^	18%	45%
Neuropathy^a^	5%	9%
G-CSF use	24%	18%
Dose reductions	25%	29%
Dose delays	22%	49%
Treatment discontinuation due to toxicity	3%	5%

*PFS* Progression-free survival, *OS* Overall Survival, *AE* Adverse Event, *G-CSF* Granulocyte Colony Stimulating Factor

^a^Only Grade 3 and 4 AE are reported here

In addition to effectiveness, our study sought to ameliorate the degree of AEs reported with standard dosing eribulin and therefore improve tolerability. Secondary end points demonstrated a lower degree of neutropenia and peripheral neuropathy. The EMBRACE trial reported grade 3 or 4 neutropenia in 45% of patients with eribulin, while our study found only 18% of patients developed this serious complication. In addition, 5% of patients in our study developed grade 3 or 4 neuropathy, while 9% were reported in EMBRACE. Overall, serious AEs were lower in number in our study, which is also supported by observed lower frequency of dose delays and dose reductions (22% and 23%, respectively). Our correlative data also shows evidence of anti-angiogenic activity of metronomic dosing.

Our study has several limitations. This was a small phase II study and does not have the power of the EMBRACE trial to compare toxicities and efficacy. Late line therapy always involves a diverse mix of patients, and this small and non-randomized trial does not fully account for this diversity. Selection bias may also play a role in our study, as patients who are willing to undergo study procedures may have better performance status or fewer clinical co-morbidities. While our correlative data showed some promise, it is limited significantly in attrition of samples during course of the study. In addition, measurement of CECs, CEPs and Apoptotic CECs is not standardized, and quantification methodology is varied across studies. Despite this, our correlative data are supportive of the hypothesis that metronomic dosing of eribulin imparts an anti-angiogenic mechanism. This hypothesis will need further validation with larger studies involving more patients. Similarly, our findings of improved therapeutic index, better tolerability and similar efficacy merit confirmation in a larger study. Given side effect profile, alternative dosing and schedules of eribulin are currently being explored [21]. Our study shows that eribulin given in a metronomic fashion, low dose has the potential to improve quantity and QOL in more MBC patients than can tolerate standard dosing.

## Conclusion

For treatment of MBC, the primary goal is to balance treatment efficacy with toxicity. In our study, we demonstrated that an altered dosing and schedule regimen of eribulin is not only effective when compared to standard dosing eribulin therapy, but also more tolerable in terms of toxicity, dose delays, and reductions. Our study found similar outcomes for patients undergoing metronomic dosing and approved dosing as reported by the EMBRACE trial. The impact of this finding could improve tolerability for many patients with MBC, allow a longer duration of therapy, prolong disease stability, and substantially improve QOL. Our phase II experience shows promising activity across all subtypes of metastatic cancer, as well as greater tolerability. The clinical implications of this study warrant further investigation for practical application.

## Supplementary Information

Below is the link to the electronic supplementary material.

Supplementary file1 (DOCX 19 KB)
