# Supplementary material for: Single arm, phase two study of low-dose metronomic eribulin in metastatic breast cancer
Source: Breast Cancer Res Treat. 2021 Apr 2;188(1):91–9. doi: 10.1007/s10549-021-06175-x (PMC8233258; doi:10.1007/s10549-021-06175-x)
Supplement: Supplementary file 1 — Supplementary file1 (DOCX 19 KB) [file 10549_2021_6175_MOESM1_ESM.docx]

**Table 1: All Adverse Events**

| **Toxicity** | **All Grades**  **n(number of individual patients)** | **Grade 3**  **n(number of individual patients)** | **Grade 4**  **n(number of individual patients)** |
| --- | --- | --- | --- |
| Alopecia | 1(1) | 0(0) | 0(0) |
| Anal fistula | 1(1) | 1(1) | 0(0) |
| Anemia | 1(1) | 1(1) | 0(0) |
| Aspartate aminotransferase increased | 1(1) | 1(1) | 0(0) |
| Blood and lymphatic system disorders - Other Lymphocytopenia | 1(1) | 1(1) | 0(0) |
| Confusion | 1(1) | 1(1) | 0(0) |
| Diarrhea | 1(1) | 1(1) | 0(0) |
| Facial pain | 1(1) | 0(0) | 0(0) |
| Fall | 1(1) | 1(1) | 0(0) |
| Fatigue | 4(4) | 3(3) | 0(0) |
| Febrile neutropenia | 3(3) | 3(3) | 0(0) |
| Gastrointestinal disorders - Other dehydration/malnutrition | 1(1) | 1(1) | 0(0) |
| Hypercalcemia | 1(1) | 0(0) | 1(1) |
| Hyponatremia | 1(1) | 1(1) | 0(0) |
| Infections and infestations - Other Infected skin lesion on chest | 1(1) | 1(1) | 0(0) |
| Investigations - Other generalized weakness | 1(1) | 0(0) | 0(0) |
| Lymphocyte count decreased | 3(2) | 2(2) | 1(1) |
| Muscle weakness lower limb | 1(1) | 1(1) | 0(0) |
| Myocardial infarction | 1(1) | 1(1) | 0(0) |
| Nausea | 3(2) | 2(1) | 0(0) |
| Neutropenia | 13(10) | 9(7) | 4(4) |
| Obstruction gastric | 1(1) | 1(1) | 0(0) |
| Pain | 2(2) | 1(1) | 0(0) |
| Pericardial effusion | 2(1) | 2(1) | 0(0) |
| Peripheral neuropathy | 3(3) | 3(3) | 0(0) |
| Thrombocytopenia | 1(1) | 1(1) | 0(0) |
| Respiratory failure | 1(1) | 0(0) | 1(1) |
| Respiratory, thoracic and mediastinal disorders - Other COPD, Dyspnea | 1(1) | 1(1) | 0(0) |
| Respiratory, thoracic and mediastinal disorders - Other pleuritic chest pain | 1(1) | 1(1) | 0(0) |
| Seizure | 1(1) | 0(0) | 0(0) |
| Sepsis | 1(1) | 0(0) | 1(1) |
| Upper respiratory infection | 1(1) | 1(1) | 0(0) |
| Urinary tract infection | 1(1) | 1(1) | 0(0) |
| White blood cell decreased | 6(5) | 4(4) | 0(0) |

**Table 2: Biomarker levels at various timepoints**

| **Timing** | **CECs /µL** | | | **CEPs / µL** | | | **Apoptotic CECs (%)** | | |
| --- | --- | --- | --- | --- | --- | --- | --- | --- | --- |
|  | **Mean** | **Median** | **Range** | **Mean** | **Median** | **Range** | **Mean** | **Median** | **Range** |
| **Baseline** (n=59) | 4.56 | 3.42 | 1.15-12.45 | 0.12 | 0.06 | 0-0.52 | 29% | 28% | 0-69% |
| **Cycle 2** (n=54) | 5.43 | 4.14 | 1.19-20.67 | 0.22 | 0.07 | 0-2.81 | 24% | 22% | 0-66% |
| **Cycle 4** (n=31) | 5.21 | 4.73 | 0.86-13.84 | 0.13 | 0.06 | 0-0.65 | 25% | 25% | 0-59% |
| **Cycle 6** (n=16) | 6.48 | 5.71 | 2.35-25.08 | 0.15 | 0.08 | 0-0.6 | 30% | 27% | 3-62% |
| **EOS** (n=24) | 5.09 | 4.49 | 1.79-11.06 | 0.08 | 0.05 | 0-0.38 | 20% | 15% | 0-64% |

**Table 3: CAIX levels at various timepoints**

| **Timeline** | **CAIX (pg/mL)** | | |
| --- | --- | --- | --- |
|  | **Mean** | **Median** | **Range** |
| **Baseline** (n=61) | 125 | 89 | 12.7-1068.2 |
| **Cycle 2** (n=55) | 121 | 94.9 | 22.6-410.6 |
| **Cycle 4** (n=33) | 107.6 | 85.4 | 37.7-293.5 |
| **Cycle 6** (n=18) | 135.9 | 91.3 | 40.9-657.6 |
| **EOS** (n=28) | 196.4 | 77.4 | 23.5-1534.3 |
